# Citizen science mitigates the lack of distributional data on Nigerian birds

**DOI:** 10.1002/ece3.11280

**Published:** 2024-04-16

**Authors:** Talatu Tende, Iniunam A. Iniunam, Samuel T. Ivande, Adewale G. Awoyemi, Bello A. Danmallam, Abubakar S. Ringim, Longji A. Bako, Fatima J. Ramzy, Nanchin W. Kazeh, Arin Izang Izang, Panshak S. Kumdet, Joseph I. Ibrahim, M. Abubakar Haruna, Kevin Eyos, Ezekiel D. Iki, Adams A. Chaskda, Ulf Ottosson

**Affiliations:** ^1^ Department of Zoology, A. P. Leventis Ornithological Research Institute University of Jos Laminga Nigeria; ^2^ Forest Center International Institute of Tropical Agriculture Ibadan Nigeria; ^3^ Biodiversity Conservation and Education Unit, Centre for Arid Zone Ecology Federal University Dutse Dabawa Jigawa Nigeria; ^4^ School of Education Federal College of Education (Technical) Potiskum Yobe Nigeria; ^5^ Becheve Nature Reserve/Nigeria Conservation Foundation Cross River Nigeria

**Keywords:** bird clubs, bird enthusiasts, citizen science, environmental awareness, human‐nature interactions

## Abstract

Citizen science projects are expanding globally, with the African continent, particularly Nigeria, registering significant growth. Here, we document and analyse novel operations of the Nigerian Bird Atlas Project (NIBAP), 2015–2022. This project has employed the use of ornithologists, mainly trained at the A. P. Leventis Ornithological Research Institute (APLORI) located in Jos, Nigeria, and its 28 bird clubs established across Nigeria to enlist 827 bird enthusiasts that contribute regular and near real‐time data about bird distribution and relative abundance in the country. Interestingly, NiBAP has recorded about 75% of the bird species known from Nigeria in only about 50% of Nigeria's total surface area, including 39 nationally threatened species. The Common Bulbul *Pycnonotus barbatus*, Laughing Dove *Spilopelia senegalensis*, and Grey‐backed Camaroptera *Camaroptera brevicaudata* were the most commonly recorded species, while Amurum Forest Reserve, Rennajj Fish Farm, and Obudu Cattle Ranch were the most surveyed sites during the period. Thus, our approach reveals how to increase involvement of nature enthusiasts, ornithologists, and a regional research institute to build local capacity and contribute rich information necessary to alleviate the lack of distributional data about Afrotropical avifauna. We strongly recommend our approach to boost other citizen science projects across Africa and beyond to address the huge lack of biodiversity data, create public awareness, and foster conservation education.

## Graphical abstract

Citizen science mitigates the lack of distributional data on Nigerian birds. Citizen scientists achieve this through the informal training provided by the A. P. Leventis Ornithological Research Institute. This training also results in positive change in attitude towards environmental conservation and awareness.

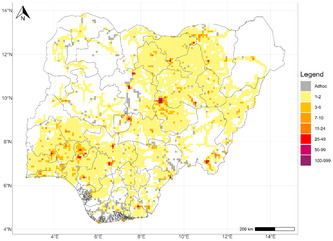

## 1 INTRODUCTION

A bird atlas is an ornithological work that seeks to provide data on the distribution and relative abundance of bird species over space and time. Such information is necessary to determine the impact of anthropogenic activities (e.g., urbanisation, human population expansion, and farming practices) on species distribution and environmental dynamics (Gibbons et al., 2007). The atlas concept was first developed to map the distribution of the British flora in the late 1950s (Perring & Walters, 1962) and was adopted for the British birds in the late 1960s (i.e., Atlas of the Breeding Birds of the Midlands, Lord & Munns, 1970 and Atlas of the Breeding Birds in Britain and Ireland, Sharrock, 2010). Today, there are at least 272 bird atlas projects with standardised protocols in 50 countries across the world (Dunn & Weston, 2008). Despite variations in methodologies, all bird atlas protocols strive to collate data relating to the presence and abundance of birds over large spatio‐temporal scales and analyse such data to determine the state of the environment (Danielsen et al., 2014; Pocock et al., 2018).

A crucial feature of an atlas project is the engagement and contribution of data by members of the public through field surveys, an approach termed citizen science (Kobori et al., 2016). Citizen science data aim to expand the understanding of species distribution as well as their life histories, and provide insights into population size and trends, which are vital for the conservation of species, particularly those of conservation concern (Dickinson et al., 2010; Sullivan et al., 2017). In the early days of ornithological study, Britain and Ireland pioneered the use of a vast network of volunteer contributors for their bird atlas projects. One of the first such efforts, conducted in 1952, marked a significant milestone in the field by implementing a grid system for the first time aimed at documenting the breeding distribution of 30 avian species throughout the United Kingdom (Norris, 1960). In recent years, the citizen science approach has received increasing attention and is growing rapidly across the world, contributing valuable data on which several bird conservation programmes and initiatives are now based (de Sherbinin et al., 2021).

Although citizen science projects have the capacity to contribute huge amounts of data, they are lacking in most biodiversity‐rich regions (Ringim et al., 2022; Squires et al., 2021). Citizen science is thus a new and growing concept in Africa (Barnard et al., 2017). A review by Young et al. (2019) revealed that of the 82% of natural heritage programmes that participate in environmental regulatory reviews, 88 and 52% mainly depend on citizen science data in Canada and the United States, respectively. Sullivan et al. (2014) and Ringim et al. (2022) showed how citizen scientists contribute large datasets in near real‐time. Aceves‐Bueno et al. (2015) made a comparison and found that data collection through citizen science is more cost‐effective than traditional approaches that are mainly based on experts and project‐related means, and have a high potential for citizen engagement. Despite these gains, the validity of the data collected by citizen scientists, most of whom are not experts, but mainly enthusiasts/volunteers, is still a subject for debate. This is a challenge that many of the new atlas projects now aim to tackle, as strong taxonomic skills are a baseline for this kind of endeavour (Bonter & Cooper, 2012; Kosmala et al., 2016). Interestingly, some atlas projects are now demonstrating sufficient quality (Stuber et al., 2022) useful to inform government policies regarding biodiversity conservation (Ruiz‐Gutierrez et al., 2021).

The first bird atlas project in Africa, built on the Birdmap protocol, the Southern African Bird Atlas Project (SABAP1), started in 1986 (Harrison & Cherry, 1997; Lee et al., 2022), almost three decades after that of Britain (Sharrock, 2010), covering most countries in southern Africa, where rapid growth in human population impacts highly on the environment. The SABAP1 resulted in the publication of the atlas of southern African birds (Harrison & Cherry, 1997), already showcasing the significance of citizen science in improving knowledge useful for promoting the conservation of African avifauna. The second bird atlas project (SABAP2) started in 2007 to build on the successes recorded earlier, expanding its reach by incorporating Kenya through the Kenyan Bird Map (KBM) in 2013 and Nigeria through the Nigerian Bird Atlas Project (NiBAP) in late 2015. Although the exact cause of this delayed start of bird atlasing in Eastern and Western Africa is unknown, it may relate to the lack of ornithological capacity in these regions relative to Southern Africa (Beale, 2018; Cresswell, 2018).

The NiBAP was started when different institutions agreed to collaborate on the project to map the distribution of Nigerian birds and create nationwide awareness of environmental issues in Nigeria by engaging the public in the project. The Percy FitzPatrick Institute of African Ornithology (PFIAO) at the University of Cape Town in South Africa provides technical support to NiBAP, drawing on the lessons learned from SABAP 1 and SABAP 2. The A. P. Leventis Ornithological Research Institute (APLORI, Nigeria) leads the project in collaboration with the Nigerian Conservation Foundation (NCF).

In this paper, we describe our experiences starting and building one of the largest and most successful citizen science projects in terms of participation and coverage in West Africa. Here, we show how our novel methods of robust citizen scientist engagement have been used to achieve greater coverage. We specifically (i) describe the foundational process of identifying, mapping, and convening pre‐existing local capacity for ornithology and birdwatching in Nigeria; (ii) describe the recruitment, capacity development, and growth of citizen scientists in Nigeria through bird clubs; and (iii) assess the impacts of this growing birdwatching and citizen science community on data generation and survey coverage for the NiBAP.

## 2 METHODS

### 2.1 Historical background

In 2002, APLORI was established to strengthen ornithological research in West Africa (Mwansat et al., 2011), an ornithologically diverse region that is relatively understudied due to a lack of local capacity (Beale, 2018; Cresswell, 2018). A key aim of the institution was that graduates from the institute would influence conservation activities, policies, and decisions within their region and beyond. This pioneering institution is a biological conservatory affiliated with the University of Jos in central Nigeria. From inception to 2023, APLORI has graduated 141 students from four West African countries, including Nigeria (133), Ghana (12), Sierra Leone (2), and Liberia (4). These students were awarded a Master's degree in conservation biology (APLORI, 2021). A good number of APLORI graduates have furthered their education by pursuing doctorates, with 25% already having completed their studies and 24% currently enrolled (APLORI, 2021). Perhaps, even more noteworthy is the fact that about 90% of these graduates continue to make meaningful contributions to biodiversity conservation through a variety of roles in research and educational institutions, government, and non‐governmental organisations (APLORI, 2021).

APLORI graduates, widespread across Nigeria, provide critical linkages for NiBAP, which began with a team of three APLORI‐related individuals (hereafter, the “project team”) in late 2015. The project team was saddled with the responsibilities of creating awareness about the project as well as dedicated data collection. The project team scheduled atlas excursions in 2016, in large part based on a distribution map of APLORI graduates, asking these graduates to learn the atlas protocol by participating in atlas expeditions and encouraging them to create and foster participation in local bird clubs. With many of these graduates now based at or affiliated with universities across Nigeria, the project team also delivered seminars to create awareness about the bird atlas project and bird conservation in Nigeria. These seminars encouraged enthusiastic attendees to sign up for guided bird walks by knowledgeable ornithologists and non‐experts, who live nearby. The resident ornithologists were also encouraged to lead at least one bird walk per month. Enlisted members were also encouraged to create social media platforms, for example, WhatsApp and Facebook groups, to facilitate organisation and communication about outings and help with bird identification. In November 2017, the first project workshop was held in Jos, where 23 participants, including APLORI graduates, bird and nature enthusiasts were introduced to the project and its protocol. These workshop participants and other enthusiasts who later received training from the project team quickly rose to the position of leadership in bird clubs and regional atlas teams that NiBAP formed around the nation (see Ringim et al., 2022).

Bird club activities revolved around ornithologists within the groups, ensuring that newly engaged participants learned new birding skills as well as the importance of birds at improving the environment and human health. During club outings, enthusiasts are introduced to new bird species and the role they play in the environment. They are also taught the bird atlasing protocols using the BirdLasser application (Lee & Nel, 2020). Individuals who demonstrate great enthusiasm are eventually recruited into one of the three Atlas teams. This means that these individuals are comfortable working in the field for long hours, covering several kilometres, and recording birds. This has eventually led to over one hundred and twenty‐eight individuals actively participating in atlas expeditions (Figure 2).

### 2.2 Nigerian bird atlas protocol

The NiBAP protocol was adopted from SABAP2. The protocol is based on grid cells, with each covering 5′ × 5′ of latitude and longitude, translating to *c*. 9 km × 9 km, known as a pentad. A mobile application called BirdLasser is designed to aid data collection using this protocol. The standard protocol involves devoted birdwatching for a minimum of 2 h, during which birds are recorded in the order they are seen/heard, with the option to keep a record of abundance, as well as information related to bird morphometrics, activities, and habitat types. The strategies adopted by all the atlas teams and bird clubs are that at least an unskilled bird enthusiast is paired with a formally trained ornithologist during bird atlas expeditions. This enhances skill transfer, motivation, a sense of community, data quality control, and the sharing of socio‐cultural information (Parrish et al., 2019; Peter et al., 2019; Turrini et al., 2018). They are also expected to visit as many habitats as possible within a pentad. The atlasers eventually review the data that has been collected before submitting it to the NiBAP database using the BirdLasser application. Observations can also be submitted as ad hoc or incidental observations if <2 h are spent in a pentad within 5‐consecutive days (120 h). A unique feature of the NiBAP protocol is that it is a “living” atlas, meaning that it is not a snapshot atlas restricted to seasons or time periods but is continuous all year round (Tende et al., 2016).

### 2.3 Data vetting, verification, and structure of the NiBAP

The Regional Atlas Committee (RAC), composed of some experienced members of the NiBAP management team at APLORI, ensures all data collected and entered into the database are vetted. An entry is flagged as out of range (ORF) if it is sighted outside of its known range and/or season of the year, or if the database lacks entries in the vicinity of the current pentad. Flagged entries involve an additional process of contacting the people who submitted them for clarification before acceptance into the database that is hosted in South Africa. The RAC rejects any entry that is unclarified or questionable and follows up with expert verification in relevant pentads.

### 2.4 Data collection and analyses

We extracted data, including the number of pentads visited and bird species recorded from December 2015 to December 2022. We also made an inquiry into the historical records of the 28 bird clubs to extract information on the number of bird enthusiasts as compared to ornithologists since the inception of the various bird clubs. We used ArcGIS to visualise the spatial distribution of ornithologically related scientists across Nigeria. We did this for both birding enthusiasts and trained ornithologists. We also explored the data contributed to the project by citizen scientists. We estimated the density of citizen scientist in all regions by dividing the total number of atlasers in a region by the area of the region. All statistical analyses were conducted in R (ver. 3.6.3, R Core Team, 2020).

## 3 RESULTS

**FIGURE 1:**
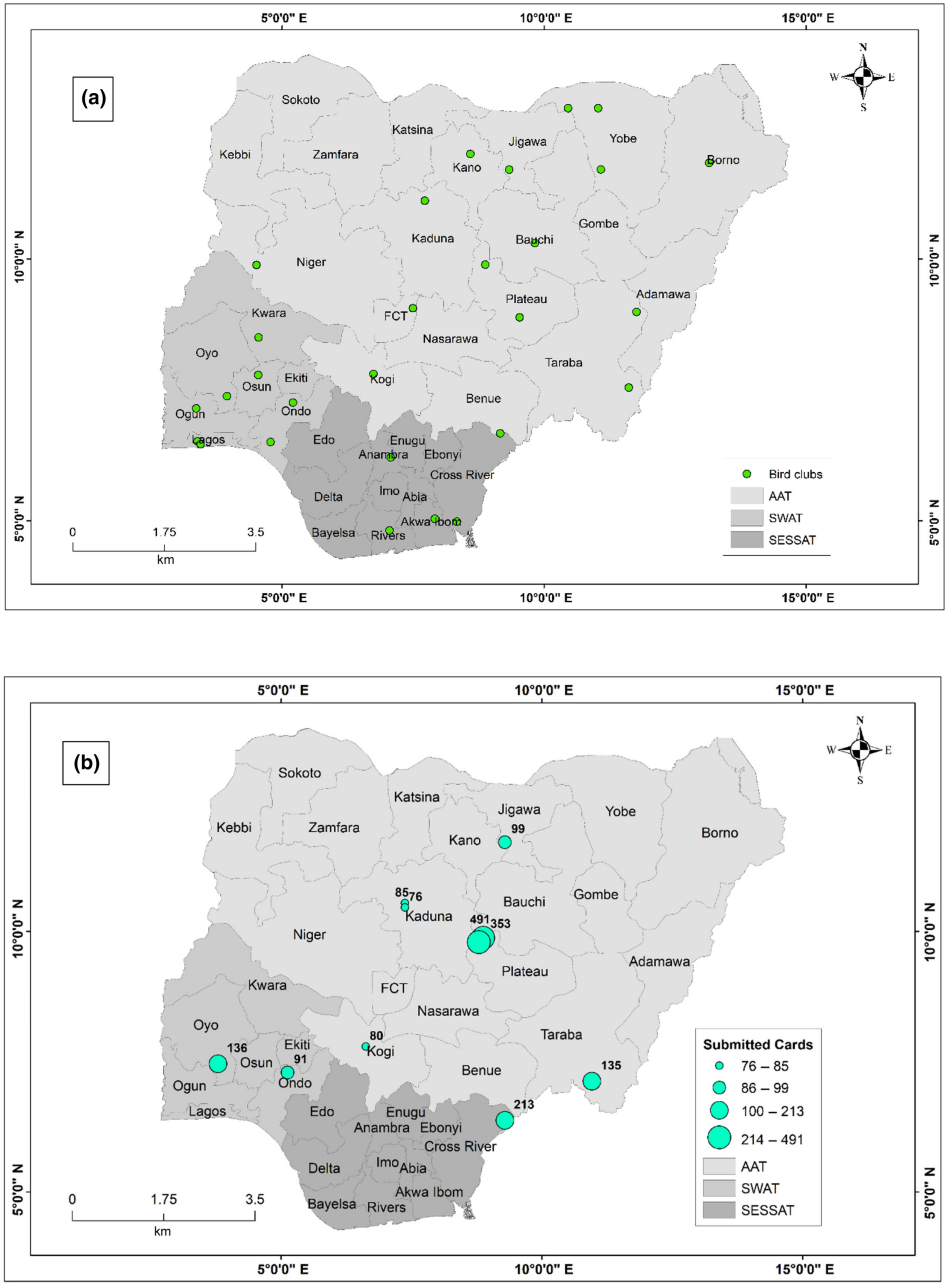
The distribution of bird clubs (a) and the pentads with the most “full protocol” submissions (b). The size of the dots in (b) represents the total number of full protocol card submissions. The AAT is the Arewa Atlas Team, SWAT is the South‐west Atlas Team and SESSAT is the South‐East South–South Atlas Team.

**FIGURE 2:**
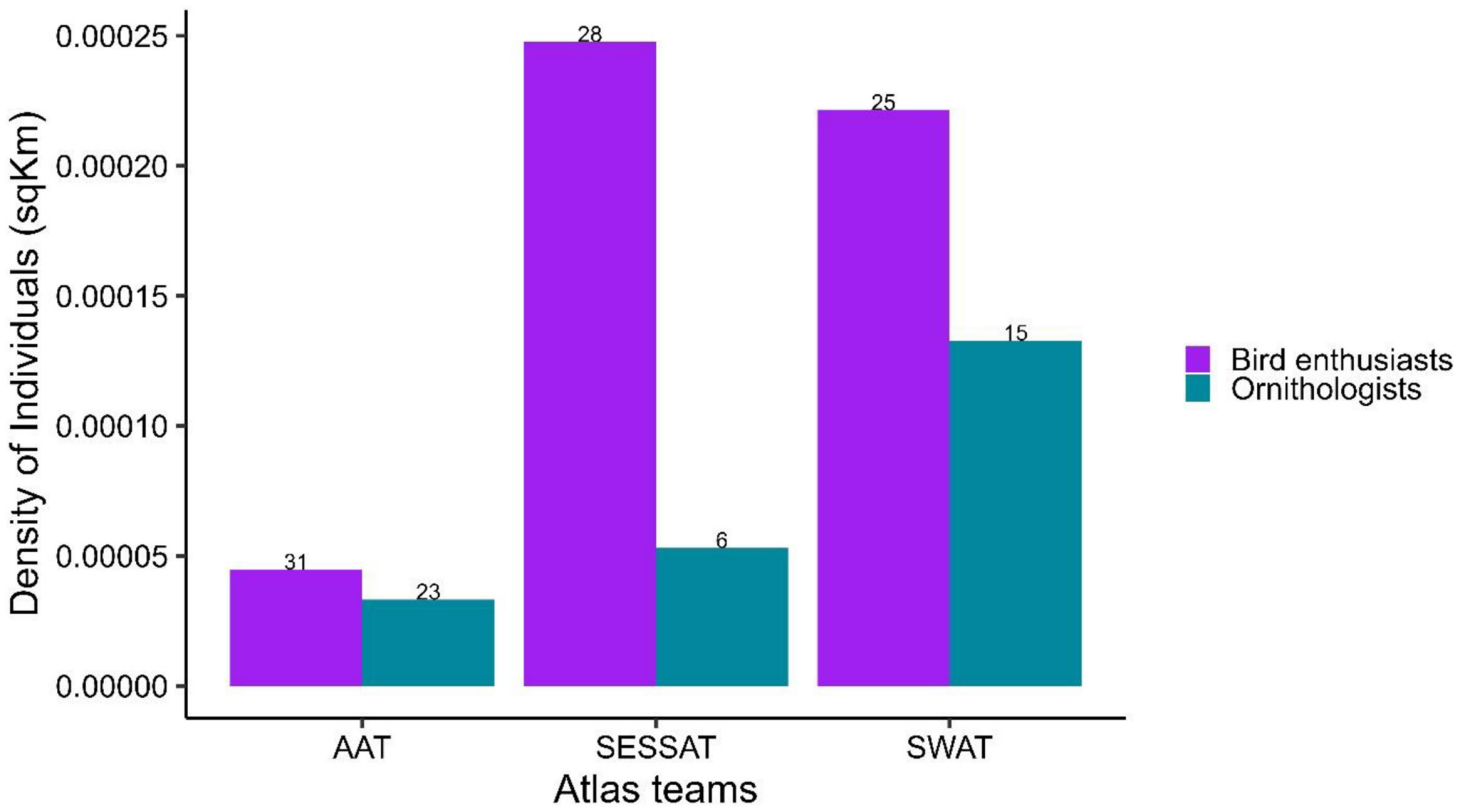
The variation in the density (*y*‐axis) and number (top of each bar) of bird enthusiasts and ornithologists in the different bird atlas teams in the Nigerian Bird Atlas Project from December 2015 to December 2022.

From our distribution data, the bird clubs are distributed across the country (Figure 1a), with enthusiasts dominating the bird clubs when compared to the trained ornithologists (Figure 2). These bird clubs enlisted a total of 827 citizen scientists between 2016 and 2022. The clubs are supported by the three regional Atlas teams, including the Arewa Atlas Team (AAT), the Nigerian Southwest Atlas Team (N‐SWAT), and the South‐East South–South Atlas Team (SESSAT). There is a higher density of atlasers in the SESSAT and SWAT compared to AAT (Figure 2).

**FIGURE 3:**
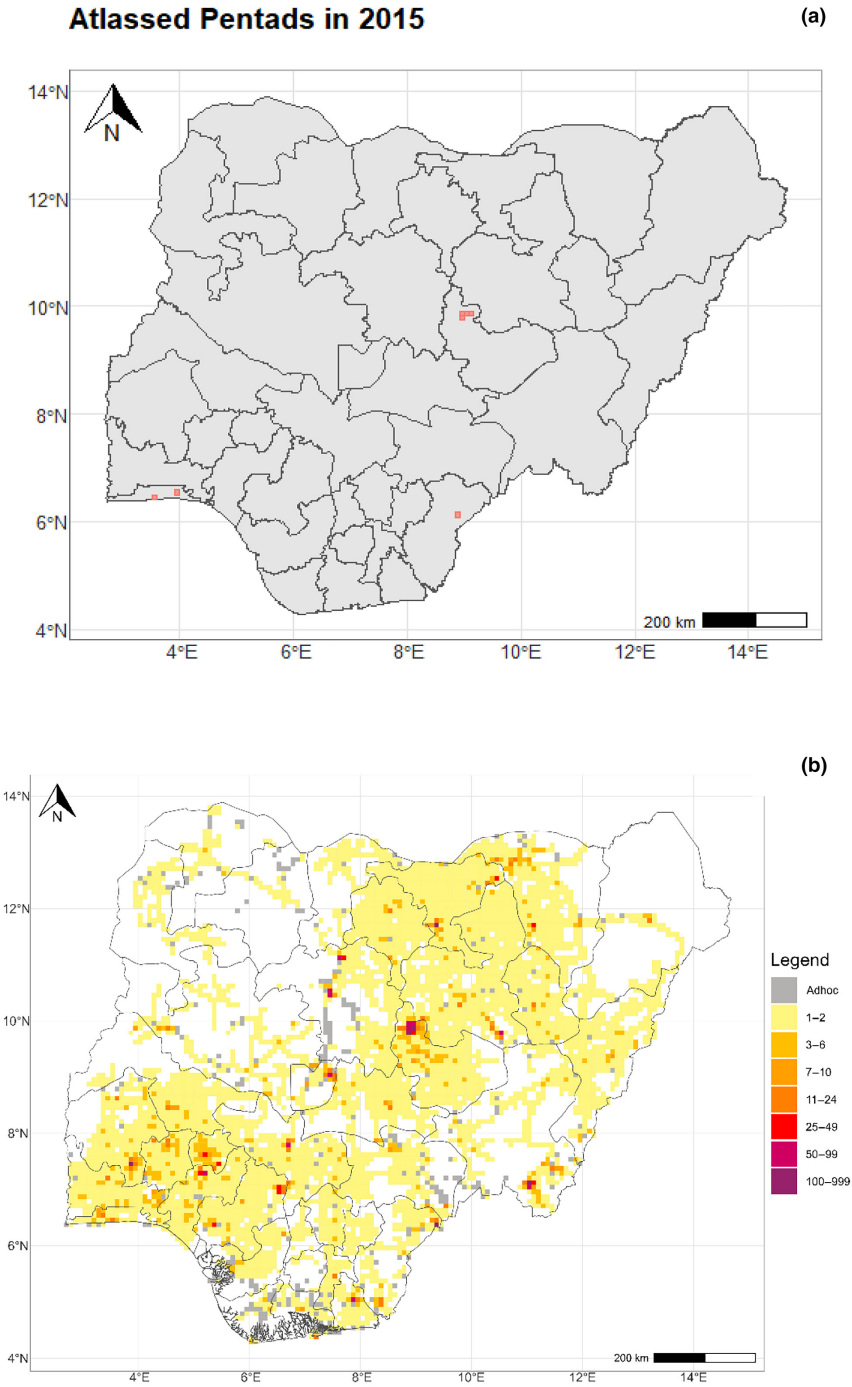
Progress of pentad coverage (pentads) by the Nigerian Bird Atlas Project from 2015 (left) to 2022 (right). The darker colours on the pentads in the 2022 map show the intensity of atlasing on those pentads.

**FIGURE 4:**
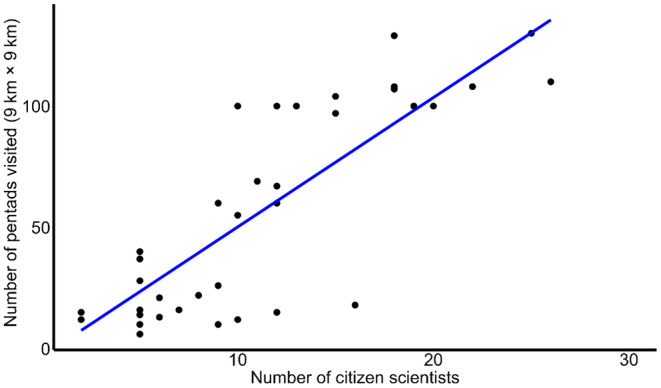
The relationship between the numbers of citizen scientists and pentads covered by the Nigerian Bird Atlas Project from December 2015 to December 2022. The blue line shows a positive trend that as number of citizen scientists increased, the number of pentads visited also increased.

At the inception of the project in December 2015, only seven pentads were covered, compared to 8709 by December 2022 (Figure 3). This coverage accounts for about 79% of the total pentads (*c*. 11,000) found in Nigeria. We also found that as the number of citizen scientists increased, more pentads were covered across the country (Figure 4). Despite this progression, there are still gaps in some areas of the country, particularly in the northwest and northeast (Figure 3).

Out of these 8709 pentads visited by citizen scientists, 5898 have been atlased comprehensively and submitted as full protocol 9306 times, while the rest (2811) were submitted as ad hoc. Cumulatively, this yielded 308,504 bird records (bird entries). Overall, 719 bird species have been recorded, accounting for 76% of the number of known bird species in Nigeria (cf. African Bird Club, 2023).

**FIGURE 5:**
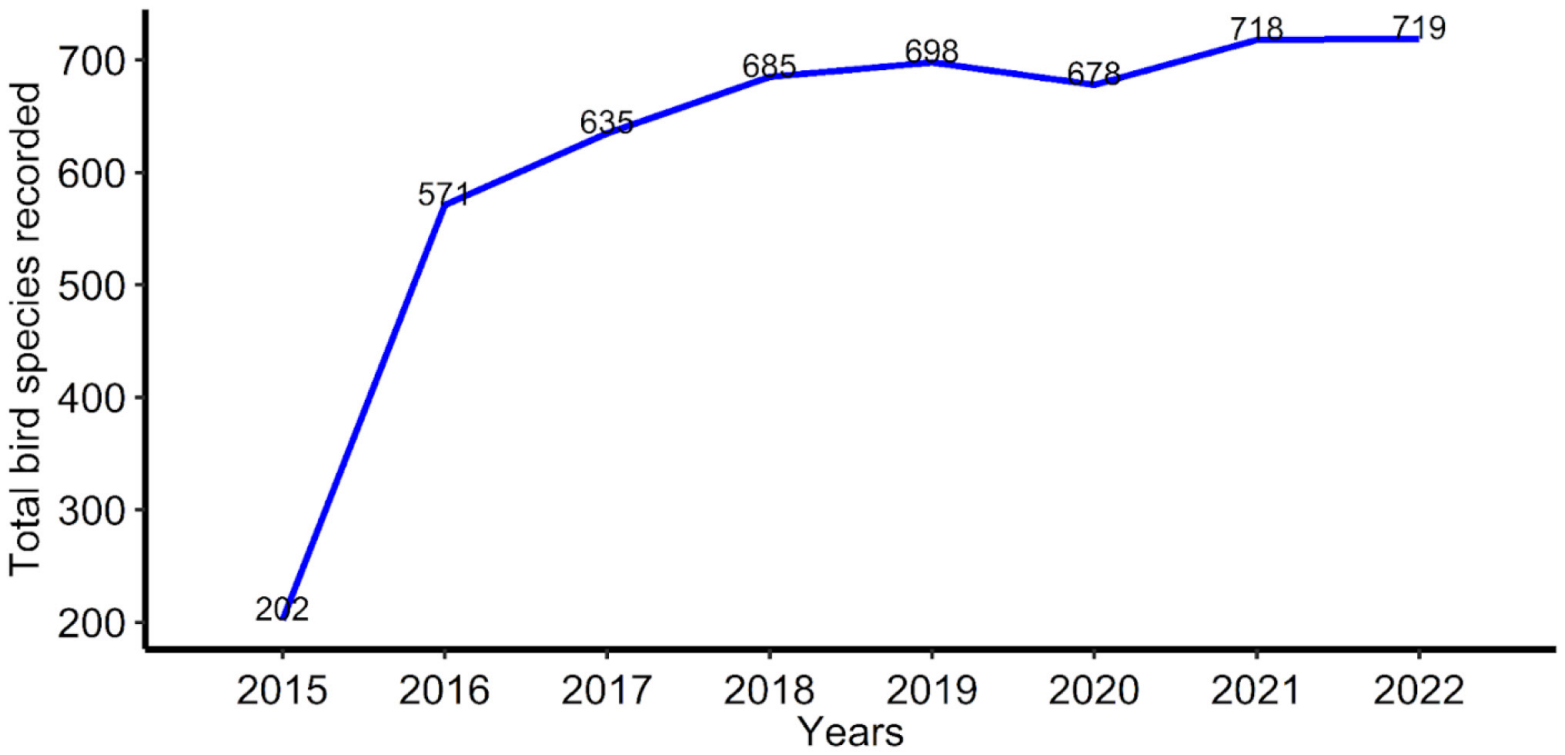
The temporal progression of bird species richness recorded by the Nigerian Bird Atlas Project from December 2015 to December 2022.

**FIGURE 6:**
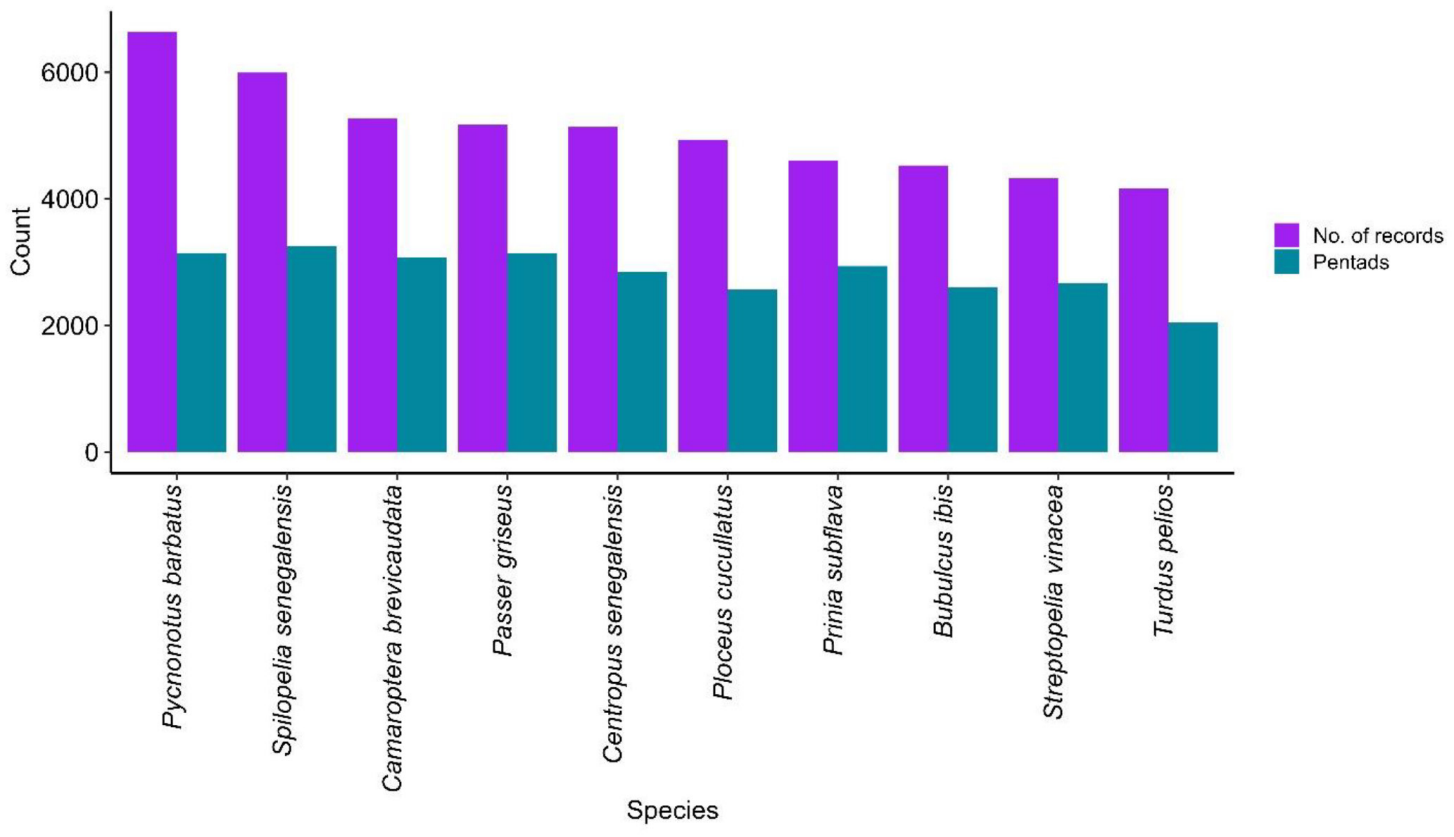
The 10 most recorded species and counts across pentads recorded by the Nigerian Bird Atlas Project between December 2015 and December 2022.

Figure 5 shows the trend in the number of bird species recorded during the seven‐year period (December 2015–2022). From both ad hoc and full protocol submissions, the five birds with the highest number of records are Common Bulbul *Pycnonotus barbatus* (6638), Laughing Dove *Spilopelia senegalensis* (5996), Grey‐backed Camaroptera *Camaroptera brevicaudata* (5267), Northern Grey‐headed Sparrow *Passer griseus* (5171), and Senegal Coucal *Centropus senegalensis* (5135) (Figure 6). The three most atlased pentads are those located within the Amurum Forest Reserve (APLORI), Rennajj Fish Farm, and Obudu cattle ranch, with 491, 353, and 213 full protocol cards submitted, respectively (Figure 1b).

## 4 DISCUSSION

Our results reveal a burgeoning citizen science initiative used to map the distribution of birds in Nigeria. Relative to other countries such as the United Kingdom (Butterfly Monitoring Scheme), the United States (Northern American Breeding Bird Survey), or South Africa (SABAP2) that already had existing protocols for several decades (Brereton et al., 2019; Sauer et al., 2013; Underhill et al., 2017), this kind of citizen science approach started late in Nigeria (December 2015). This may be due to several factors, one of which is the lack of local experts in the area (Cresswell, 2018). With an ever‐increasing human population in Nigeria (Aliyu & Amadu, 2017), there is an increasing pressure on natural areas and habitats. This has resulted in the destruction of nature and the associated biodiversity. With inadequate funding for research and development, scientific output in Nigeria, and by extension, many African countries, is very low relative to other regions of the world (Awoyemi & Ibáñez‐Álamo, 2023; Cresswell, 2018). This suggests that some species could well become locally extinct before being discovered, as an understanding of species distribution is a key prerequisite to any successful conservation action. This pattern of regional extinction events is not uncommon in Africa. However, our study has revealed how to partially ameliorate the shortage of scientific research and useful data through the NiBAP.

Although comparing the output of the NiBAP initiative to those from traditional research conducted in the area is out of scope of this study, the NiBAP data were generated in real‐time and at a relatively reduced cost, as shown by similar previous studies (Burgess et al., 2017; Kullenberg & Kasperowski, 2016). From a conservation standpoint, it is also imperative to say that the NiBAP protocol could be applied to other understudied taxa in Nigeria, such as insects, amphibians, and reptiles, as previous studies have highlighted (e.g., Awoyemi & Ibáñez‐Álamo, 2023).

Despite the delayed start of bird atlasing in Nigeria, the timing appears to be an important factor that has enhanced citizen science and NiBAP coverage in the area. This is because, although NiBAP commenced almost 13 years after the establishment of APLORI, the lag allowed the training of over 100 expert ornithologists well‐spread across the different habitats and vegetation zones in Nigeria. We are not aware of any other citizen science initiatives harnessing such a robust synergy between experts and non‐experts to win support, collect data, and consequently promote the conservation of birds in Africa. While such citizen science initiatives occur in other countries such as South Africa (Lee et al., 2022) and Kenya (Wachira et al., 2015), they are not as organised, productive, and consistent as the NiBAPs. Thus, NiBAP demonstrates the viability and success of an ornithological citizen science project in Africa, which is useful for replication in other countries. It is also worthy of note that the two leading countries (South Africa and Nigeria) in bird atlasing in Africa have ornithological research institutes, the PFIAO and APLORI, respectively. This justifies the investment in and productivity of these research institutes and the need for more in Africa, at least in specific regions such as Northern, Central, and Eastern Africa, where similar institutes are currently lacking. Although situated in Nigeria, APLORI graduates are distributed in four of the 16 West African countries, as shown in our study. If well‐harnessed, the APLORI graduates in those countries are useful agents of change regarding the extension of the NiBAP bird atlasing protocol to those countries, which could also be useful for decision‐making by potential donors.

The use of APLORI graduates as a foundation for the formation of bird clubs and the eventual incorporation of interested members into the atlasing teams produced remarkable results. For instance, this increased bird enthusiasts who became part of the atlasing teams made it easier for more pentads to be covered over time (Figure 4). The high density of bird enthusiasts and ornithologists registered in the south‐south and south‐western regions enhanced the widespread coverage recorded in these areas. This is further supported by the reduced pentad coverage in the northwest (e.g., Kebbi, Sokoto, and Zamfara States), where APLORI‐trained ornithologists are thinly spread. The density of atlasers in the northern region was quite low when compared to other regions, thus contributing to the reduced pentad coverage in the northern region. Furthermore, the limited pentad coverage in north‐eastern Nigeria, especially in Borno State, was more related to the Boko Haram insurgency than the availability of trained ornithologists.

Prior to the introduction of the NiBAP bird clubs, the Lekki and Ibadan Bird Clubs in South‐West Nigeria already existed (Awoyemi & Bown, 2019). The NiBAP citizen science approach has significantly increased the number of bird clubs in Nigeria to 34, 28 of which are operational (Figure 1a). Interestingly, membership in approximately 86% of the bird clubs has also grown exponentially (cf. Table S1), while the proportion of bird enthusiasts in the three atlasing teams was higher than that of ornithologists, corroborating that the formation of bird clubs and atlasing teams can promote interest in birds and conservation‐related issues. This trend relates to that found by Squires et al. (2021), who demonstrated that the BigMonth2020 event attracted many novice bird watchers. Harebottle (2020) also shows that the involvement of passionate volunteers promotes citizen science projects.

Although studies have shown that data collection by citizen scientists is prone to errors and could raise concerns about data quality (Bonter & Cooper, 2012; Kosmala et al., 2016), the vetting and verification by RAC in our case seem to overcome this important barrier, suggesting the reliability of the NiBAP database, and the need for related initiatives to adopt or maintain a similar vetting process. Another perspective is that our approach seems to win more advocates for bird conservation in Nigeria, where the appreciation of natural heritage has been low (e.g., Awoyemi et al., 2024), but it is necessary to win support for conservation in the area.

Perhaps of utmost interest is the record of *c*. 80% of the total number of bird species recorded in Nigeria. We found that as the number of citizen scientists increased, more pentads were covered, resulting in more species being recorded. The reduction in the number of recorded species in 2020 was mainly due to the lockdown measures enforced due to the COVID‐19 pandemic. Despite the pandemic, more species were recorded as the years progressed. Recording *c*.80% of bird species in only *c*.50% of pentads found in Nigeria suggests that the NiBAP initiative could even document more bird species than ever reported for Nigeria, thus updating the bird checklist for the country, which will benefit future conservation decisions.

Among species recorded to date are 39 IUCN‐listed threatened species, including three Critically Endangered, nine Endangered, thirteen Vulnerable, thirteen Near Threatened, and one Data Deficient species (cf. Appendices S1–S8). Of the total species recorded, 30 are intra‐African migrants, 94 are Palearctic migrants, and the majority, which accounts for approximately 85% of the records, are resident species. The most commonly recorded species in our database are categorised as generalists (Borrow & Demey, 2014) that could be found in different habitats, especially in urban areas, which are relatively easy to survey by our members.

In summary, in this study, we show how to enhance the potential of citizen science for data collection and the promotion of biodiversity conservation at a relatively low cost. Our generated data are useful to monitor and analyse population trends, identify areas with high or low associated bird diversity, and examine the health of various ecosystems. We have also shown how this kind of initiative trains potential local experts, which are urgently needed in Africa (Awoyemi & Ibáñez‐Álamo, 2023). For instance, some of our members have advanced in their scientific endeavours, analysing and publishing several bird‐related papers. Thus, our approach reveals the capacity development of enthusiasts into reliable citizen scientists through knowledge transfer from ornithologists and the importance of APLORI in capacity building across the West Africa sub‐region.

While we acknowledge the fact that most countries may not have such institutions like APLORI in Nigeria or PFIAO in South Africa, they can build on existing infrastructure such as the national BirdLife Partner and or academic institutions with ornithological, ecological, or conservation orientation. A combination of these institutions coupled with the bird club models can start or grow the citizen science projects. We hope that our approach will be adopted by other citizen science projects in Africa to help address the huge lack of biodiversity data, public awareness, and conservation education.

## AUTHOR CONTRIBUTIONS

**Talatu Tende:** Conceptualization (lead); methodology (equal); writing – original draft (lead); writing – review and editing (equal). **Iniunam A. Iniunam:** Conceptualization (equal); formal analysis (lead); investigation (equal); methodology (equal); visualization (equal); writing – original draft (lead). **Samuel T. Ivande:** Conceptualization (lead); methodology (equal); writing – review and editing (equal). **Adewale G. Awoyemi:** Conceptualization (equal); formal analysis (supporting); methodology (equal); writing – original draft (lead). **Bello A. Danmallam:** Data curation (lead); formal analysis (supporting); writing – review and editing (supporting). **Abubakar S. Ringim:** Writing – original draft (equal); writing – review and editing (equal). **Longji A. Bako:** Writing – review and editing (supporting). **Fatima J. Ramzy:** Data curation (supporting). **Nanchin W. Kazeh:** Data curation (equal); writing – review and editing (equal). **Arin Izang Izang:** Data curation (equal). **Panshak S. Kumdet:** Data curation (equal); writing – review and editing (supporting). **Joseph I. Ibrahim:** Writing – review and editing (equal). **M. Abubakar Haruna:** Data curation (equal). **Kevin Eyos:** Data curation (equal). **Ezekiel D. Iki:** Data curation (equal). **Adams A. Chaskda:** Supervision (equal); writing – review and editing (supporting). **Ulf Ottosson:** Conceptualization (equal); supervision (equal); writing – review and editing (equal).

## CONFLICT OF INTEREST STATEMENT

None of the authors have any conflict of interest.

## Supporting information


Appendices S1–S8.

## Data Availability

Data will be made available upon request. The data files include in Appendices S1–S8.
